# Influence of Impurities on the Pyrolysis of Polyamides^1^

**DOI:** 10.6028/jres.063A.021

**Published:** 1959-12-01

**Authors:** Sidney Straus, Leo A. Wall

## Abstract

A study has been made of the effect of drying and purification of polycaprolactam (nylon-6) on its thermal decomposition. The conclusions drawn from previous work, that a hydrolytic mechanism is at least partially operating due to the presence of water and acid polymerization catalysts, are confirmed. Purification of the material decreases the rate of degradation and the production of CO_2_, and increases the preexponential factor and the activation energy. The hydrolytic decomposition, which competes with the thermal free-radical degradation process, would be expected to produce carboxylic acids and amines, the former decomposing further to form CO_2_. The preexponential factor is increased by sample purification from 10^7^ to 10^10^ and the activation energy from 34 to 43 kcal/mole. The decomposition behavior of the purest sample is a nearly perfect example of the random decomposition of linear polymer chains. All other samples of polyamides thus far studied also appear to decompose by random or nearly random mechanisms.

## 1. Introduction

Various nylon samples were recently thermally degraded in a vacuum in order to study the mechanisms and kinetics of breakdown [1].^2^ Large variations were found in the values of the activation energies and preexponential factors, and these values were considerably lower than those found in the degradation of other polymers [2,3]. The activation energies varied from 15 to 42 kcal/mole. In addition, H_2_O and CO_2_ were found in the volatile products from the pyrolysis. These products are not readily explained on the basis of the polyamide structure of nylon. The behavior was incompatible with a pure free-radical mechanism, and the simplest explanation was that traces of absorbed H_2_O and polymerization catalysts might influence the pyrolysis to a great extent.

The present work was undertaken to study a nylon-6 sample, viscosity-average molecular weight of approximately 60,000, which previously showed considerable amounts of H_2_O and CO_2_ in the decomposition products, and to attempt to eliminate these products in the volatiles. There was a possibility that this study might yield a more thermally stable polyamide—one with a low rate of volatilization and a high energy of activation. The CO_2_ component in the volatiles is assumed to result from the decomposition of acid groups produced by hydrolytic scission. A stoichiometric reaction for this CO_2_ formation on pyrolysis of the nylon is as follows:

**Figure:**
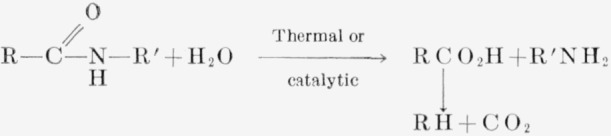


## 2. Materials

The nylon-6 sample has been described previously [1]. It was made from caprolactam by the National Aniline Company, Hopewell, Va. In addition, a nylon-66/6 sample, known as sample 4408 and described previously [4], was also used in this work.

## 3. Apparatus

The nylon-6 sample was leached, dehydrated with absolute ethanol, and subsequently dried in various ways. These leaching and drying processes are explained more fully later. The dried products were then studied in two separate sets of apparatus, as described previously [1, 5, 6, 7], by (1) identification of the various products of decomposition after pyrolysis in a tube furnace and a subsequent analysis of the lighter volatiles in the mass spectrometer, and (2) determination of the rates of volatilization of the polymer using a tungsten spring balance in an evacuated system, from which calculations were made of the activation energies. Comparisons could then be made of these results with those obtained previously on the untreated nylon-6 sample.

## 4. Experimental Procedure and Results

The CO_2_ and H_2_O collected from the thermal decomposition of the untreated nylon-6 sample are contained in the light volatiles, or “condensed” fraction, which is volatile at room temperature [1]. A breakdown of this volatile fraction in mole percent of the components is indicated in previous experimental work [1]. The CO_2_ and H_2_O components make up more than 90 weight percent of this light fraction, and this fraction comprises approximately 10 weight percent of the total volatilized part on pyrolysis at 400° C for 30 min in a vacuum.

**Figure 1:**
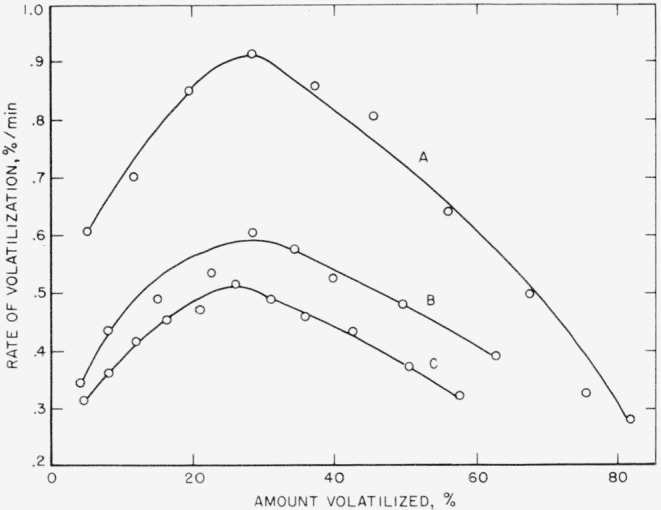
Thermal decomposition of nylon-6 at 355° C for 140 minutes. A. Untreated; B, leached in H_2_O; C, leached in H_2_O, followed by ethanol soaking.

The nylon-6 sample was first treated in the following manner in order to eliminate as much as possible any absorbed H_2_O and trace catalyst, which it is assumed are the cause of this high yield of CO_2_ and H_2_O in the light volatiles. (1) The sample was leached in distilled H_2_O for about 5 to 10 days and then dried at 110° C in the tube-pyrolysis apparatus for 1 hr while being evacuated. Elimination of any trace catalyst in the nylon sample was the chief purpose of this procedure. (2) The dried sample was then immersed in absolute ethyl alcohol for 2 days to absorb any traces of moisture from it, and the sample was again dried quickly at 110° C, as previously mentioned. Immediately thereafter, rates of volatilization at 355° C were determined in the tungsten spring apparatus. The results of these rate studies are shown in figure 1, which indicates the rate of volatilization of the sample in percent per minute as a function of the percentage of volatilization. The maximum rates of volatilization, which take place at about 25 to 30 percent volatilization, can be compared for an indication of the relative thermal stabilities. The untreated nylon sample has a maximum of about 0.9 percent/min at 355° C; leaching the sample in distilled water reduces the maximum rate of volatilization to about one-third this value. Nylon that had been leached over periods of approximately 6 months or longer had rates of volatilization that were higher than those of the untreated nylon sample. Apparently, too long a leaching period in water has a deteriorative effect, possibly due to oxidative degradation. The soaking in ethanol, as the second step, reduces the rate to almost one-half that of the untreated nvlon-6 sample. The improvement in the thermal stability is therefore almost two-fold. Moreover, it will be shown that a leached and ethanol-soaked sample that had been bottled dry at room temperature for several months had not reverted to the faster rate on standing.

The yields of light volatiles obtained on the pyrolysis of these treated nylon samples at 400° C in the tube furnace are shown in table 1. In this table and also in the other tables the yields are reported as weight percent of the volatilized portion of the polymer. In the case of the untreated nylon sample the yield of light volatiles amounts to 10.6 percent of which 7.5 percent is CO_2_ and 2.2 percent is H_2_O. The leached nylon sample indicates a yield of light volatiles of about 7.0 percent, of which 4.8 percent represents CO_2_ and 1.6 percent H_2_O. After the next step, which consists in soaking the leached nylon in dried ethanol, the yield of CO_2_ and H_2_O on pyrolysis are down to 2.7 percent of the total volatiles, which is a decrease to less than one third of that of the original yield.

At this point attempts were made to lower the rate of volatilization still further, as well as to decrease the yield of CO_2_ and H_2_O below 2.7 percent of the volatiles. Four different treatments were tried, using in all cases a nylon-6 sample that had been leached, alcohol-soaked, and then dried.

### Treatment No. 1

The nylon sample was placed in a closed tube containing *p*-dioxane and heated in an oven for 4 days at about 110° C. It was thought that the dioxane would remove traces of H_2_O in the nylon.

### Treatment No. 2

Powdered NaH was placed at the bottom of a closed Pyrex glass tube in which a glass rod seat extended up from the bottom of the tube about 10 cm. The nylon sample was placed in a smaller Pyrex tube closed at one end. This smaller tube was inserted into the NaH tube so that it rested on the glass rod seat. The nylon did not come in contact with the NaH. The larger tube was evacuated to about 10^−4^mm of Hgand then sealed off. The evacuated sealed tube was heated in an oven at about 105° C only during the 8-hr working day for the next 20 days. The sample tube remained at room temperature during the other hours of the day. NaH reacts readily with H_2_O to release H_2_.

### Treatment No. 3

Toluene and small pieces of Na were placed at the bottom of another sealed tube similar to that in treatment No. 2, and again a smaller tube with nylon sample enclosed was placed on the glass rod seat so that the sample did not come in contact with the toluene or the Na. This large tube was evacuated at 77°K and then sealed off. This tube was similarly heated for 20 days in an oven as described in treatment No. 2.

### Treatment No. 4

**Figure 2:**
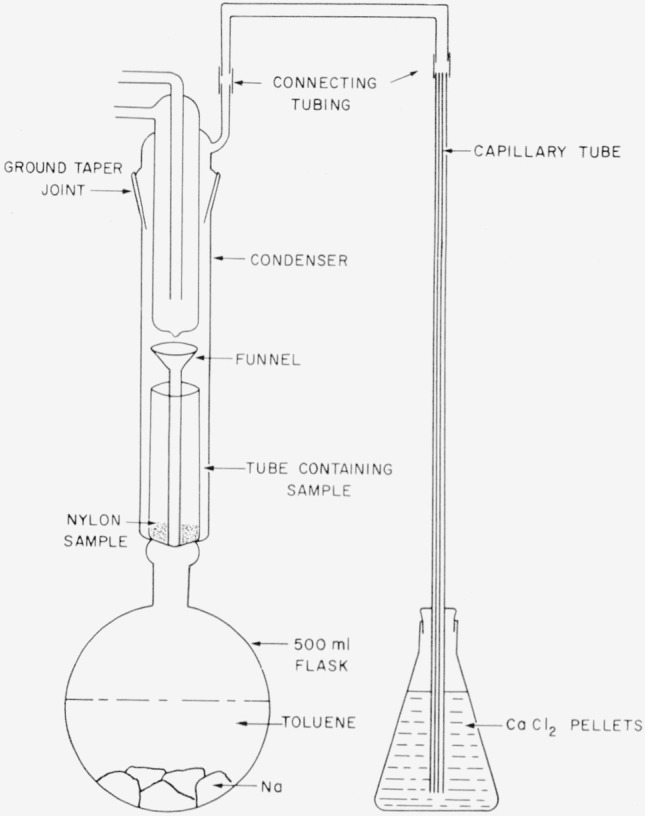
Drying system.

A 500-ml flask, resting on an electric heating mantle, was half filled with toluene and chunks of Na. Extending up from the flask was a condenser-type arrangement wherein a small test tube containing the nylon sample was held. The flask was heated at about 110° to 115° C, and the toluene evaporated into the condenser, condensed, and then was guided to drop to the bottom of the test tube containing the sample (see fig. 2). In this manner the nylon came in contact with a fresh stream of dried toluene throughout the heating. The apparatus was set up so that air was excluded, and any H_2_ gas that might be formed from the reaction of Na and H_2_O was directed out through a flask containing CaCl_2_ pellets. The nylon sample was treated this way continuously for 20 days, removed from the test tube, and dried at 110° C in a vacuum.

**Figure 3:**
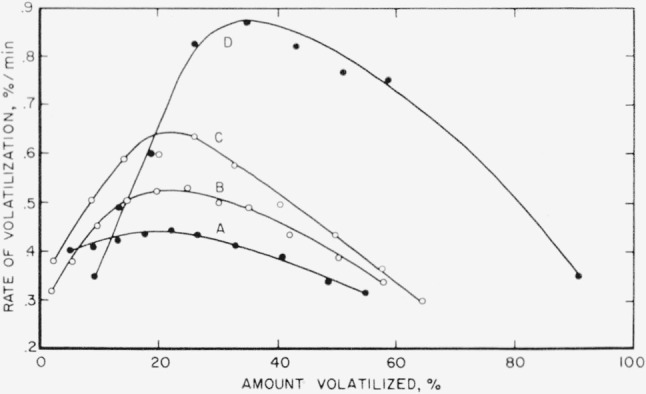
Thermal decomposition of treated nylon-6 at 355° C for 140 minutes. A, Stored wet in toluene refluxing from sodium; B, stored dry in vacuum over NaH; C, stored dry in vacuum over sodium and toluene; D, heated in *p*-dioxane.

The nylon samples, after these four individual treatments, were pyrolyzed in the rate apparatus at 355° C. The results can be seen in figure 3.

The maximum rate of volatilization of the leached and ethanol-soaked sample was slightly more than 0.5 percent/min at 355° C, as shown in figure 1. According to the curves in figure 3, the only two promising treatments were therefore the NaH dry-vacuum technique and the one wherein the nylon was continuously in contact with toluene refluxing from Na. The rate of volatilization in the NaH experiment was no improvement over the leached and ethanol-immersed nylon sample but the toluene-refluxed sample showed a further decrease in the rate of volatilization to about 0.45 percent/min. The other two experiments actually showed increases in the rate of volatilization.

These 4 treated samples were also pyrolyzed in the tube furnace at 400° C for 30 min, and with the aid of the mass spectrometer the CO_2_ and H_2_O were determined quantitatively (table 2). The total yield of light volatiles was diminished to about 6 percent in the case of the NaH treatment. On the other hand, the yield of CO_2_ and H_2_O increased over the previous low of 2.7 percent. The treatment showing the lowest rate of volatilization, storing the nylon sample wet in toluene refluxing from Na, also showed the lowest yield (1.8%) of CO_2_ and H_2_O. The relatively large amount of light volatiles obtained from samples 2,3, and 4 in table 2 results from small amounts of the solvents, toluene and dioxane, remaining in the polymer after treatment, as indicated by mass spectrometry.

It was thought that further refluxing of the toluene in the 500-ml flask over a longer period of time might still further improve the thermal stability of the nylon. Therefore, a fresh nylon sample that had been leached and alcohol soaked was continuously refluxed for a period of 76 days. The results yielded higher rates on volatilization probably because the nylon was being slowly degraded from contact with heated toluene over a long period of time.

**Figure 4:**
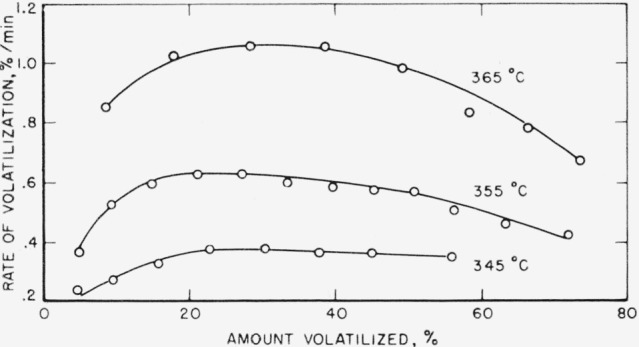
Thermal decomposition of leached and alcohol-soaked nylon-6 at various temperatures.

The treatments given the nylon-6 samples have shown an effect on the activation energies and pre-exponential factors, calculated from the rates of volatilization. Previously an activation energy of 34 kcal/mole and a preexponential factor of 4.1×10^7^[1] were obtained on the untreated nylon sample. Rates of volatilization of the nylon sample that had been leached and alcohol soaked indicated an increase in these quantities. These rates were also determined at three temperatures (fig. 4) for a treated sample that had been dried but kept bottled at room temperature for over 4 months. The maximum rate at 355° C was just slightly higher than that obtained for a similar sample that had been leached, alcohol soaked, dried, and then tested immediately. Figure 1 indicates a maximum rate of 0.52 percent/min for the latter tested sample. On the basis of the maximum rates at the three temperatures shown in figure 4, an activation energy of 41 kcal/mole was obtained. The preexponential factor was raised to 9.0×10^9^.

There was only sufficient nylon-6 sample stored wet in toluene refluxing over Na for 20 days to obtain rates of volatilization at two temperatures. The maximum rates obtained at 355° and 365° C pyrolysis were 0.44 and 0.76 percent/min, respectively. The calculated activation energy and preexponential factor based on these two rates were 43 kcal/mole and 3.2×10^10^, respectively.

A sample of the leached and ethanol-immersed nylon-6 was placed for 7 days in concentrated NH_4_OH diluted with 3 volumes of distilled H_2_O. At the end of the 7 days the nylon was dried at 110° C in the tube-pyrolysis apparatus for 1 hour. It was thought that the NH_4_OH would tend to neutralize the last vestiges of acid remaining in the nylon, thus yielding less CO_2_ in the volatiles and possibly a more thermally stable polyamide. Results on pyrolysis at 400° C for 30 min were not very conclusive. The light volatiles amounted to 8.5 percent on pyrolysis, of which CO_2_ was 3.4 percent, and H_2_O 3.1 percent, indicating an increase in these volatiles. It is obvious that treatment with NH_4_OH facilitates the hydrolytic degradation rather than prevent it by removing trace acidity.

Attempts were also made to show that by contamination of nylon with acid CO_2_ formation would increase on decomposition. A nylon sample was selected that formed much less CO_2_ and H_2_O on pyrolysis than did the nylon-6 sample. The nylon selected, nylon sample 4408 [4], was a copolymer composed of 60 percent of nylon 66 (hexamethylenediamine-adipic acid salt) and 40 percent of nylon-6 (caprolactam). It was soaked for several days in a 1.0 percent solution of orthophosphoric acid, dried, and pyrolyzed at 400° for 30 min. The untreated nylon copolymer yields 10.3 percent of the light volatiles (table 3), of which the main constituents are CO_2_ 3.7 percent, H_2_O 1.1 percent, and cyclopentanone 4.4 percent. Cyclopentanone is reported to be a decomposition product of adipic acid under certain conditions [8]. In our apparatus only a trace of cyclopentanone is produced from decomposition of adipic acid. Immersion of the nylon 4408 in dilute orthophosphoric acid has a pronounced effect on the thermal degradation reaction. The amount of CO_2_ produced was doubled, whereas the amount of cyclopentanone was decreased by a factor of 10. This result indicates that the cyclopentanone is produced not from adipic acid but directly from the polyamide. As expected, the addition of acid catalysts in the nylon sample tends to increase the yield of CO_2_ on decomposition at elevated temperatures.

## 5. Discussion

It is evident that polymerization catalyst and moisture contamination markedly affect the thermal decomposition of polyamides. The simultaneous lowering of the production of CO_2_ and the rate of volatilization by purification of the polymer indicates that hydrolytic decomposition ordinarily overshadows the thermal free-radical decomposition mechanism and accounts for the major portion of the CO_2_ observed in previous work. Although no analysis was made for amines, these products reported in other work [9] would very likely also be a result of this hydrolytic mechanism.

A completely pure polyamide of 60,000 viscosity-average molecular weight should give rise to less than 1.3 percent CO_2_ (table 2) on pyrolysis if both ends of every molecule terminate in carboxyl groups. Assuming a number-average molecular weight of 30,000 and carboxyl end-groups, one would expect a maximum CO_2_ yield of 0.3 percent. Considering the general nature of polymeric materials and their behavior on pyrolysis, a 1.3 percent CO_2_ yield may be as low as one should expect to obtain. In the case of the polyamide experiments described here, there are several possible causes for the apparent inability to purify the material to such an extent as to decrease the CO_2_ production to several tenths of 1 percent. It may well be that the last traces of moisture remain associated with peptide groups, and even in the absence of trace acids hydrolysis takes place in significant amounts in the polyamide links. There is also the likelihood that the polymers become slightly oxidized on standing prior to the experiments or that the treatments given the polymer may lead to some oxidation. Lastly, if the molecular weight distributions are actually somewhat broader than the “most probable” and high with carboxyl end groups in the low molecular weight species, 1 percent CO_2_ would be very easily accounted for.

The decrease in cyclopentanone production from the polymer containing adipate units upon contamination with acid clearly indicates that this species is produced essentially by a free-radical mechanism as an initial product from the decomposition of the polymer chain. It is apparently not produced from adipic end units or from small amounts of free adipic acid [4] initially present or formed as a result of the hydrolytic process.

The character of the rate curves in figure 4 is almost precisely the same as that for random break-down. For such a mechanism the rate, *dC/dt*, in percent or fraction of original material per unit time is approximately equal, initially, to *kL*^2^/*N* and to *kL/e* at the maximum, which for a perfect random breakdown occurs at a conversion of 26 percent [10]. The quantity, *L*, is the smallest degree of polymerization which must decompose in order to evaporate; *N* is the degree of polymerization of the starting material; and *e* is the base of the natural logarithm. In previous work [1] *L* was assumed to be 5, which corresponds in the case of polycaprolactam to a molecular weight of 565. In other words, it is assumed that breakdown occurs at the peptide links. From the variety of volatile products [1, 4] it is obvious that this is not completely true, but it allows one to make a reasonable estimate of the rate constants and hence to compare preexponential factors. The rate constant, *k*, may or may not represent a simple specific constant [1]. It clearly has concentration factors when the hydrolytic process predominates. This is a reasonable inter pretation for the very low preexponential factors found in the previous work.

The results show that the preexponential factors increase to more reasonable values for a free-radical mechanism of pyrolysis with purification of the material. Also the activation energy increased by 9 kcal/mole in the best case. The actual rate of volatilization at the temperature used in these investigations decreased by a relatively small degree since the changes in activation energy and preexponential factor tended to compensate for each other. However, the rather spectacular increase in activation energy means that at any lower temperature the thermal stability of the polymer was very markedly increased.

In polyethylene [3] and poly(*α*-methylstyrene) [2] the preexponential factors found were, respectively, of the order of 10^16^ and 10^18^. The “best” factor found in this work was 3.2×10^10^. This suggests that higher values might be attainable and leads to speculation as to how large the activation energy should be for a pure thermal free-radical mechanism of breakdown. On the assumptions that the rate of volatilization at the temperature used would be only imperceptibly decreased and that a “normal” preexponential factor is 10^13^, one estimates an activation energy of 50 kcal/mole. A high value of 10^16^ would mean 60 kcal. An activation energy between 50 to 60 kcal would, we believe, be a reasonable value for the reactions involving the pyrolysis of pure polyamides if the mechanism were purely free radical.

## Figures and Tables

**Table 1:** Products from pyrolysis of nylon-6 at 400° C for 30 min

Sample	CO_2_	H_2_O	Total light volatiles
			
	%	%	%
1. Untreated	7.5	2.2	10.6
2. Leached with H_2_O	4.8	1.6	7.0
3. Leached with H_2_O followed by ethanol soaking	2.2	0.5	6.7

**Table 2:** Products from pyrolysis of nylon-6 after treating leached and alcohol-soaked sample

Sample	CO_2_	H_2_O	Total light volatiles
			
	%	%	%
1. Stored dry in vacuum over NaH	2.6	1.3	5.9
2. Stored wet in toluene refluxing from Na	1.3	0.5	7.3
3. Stored dry in vacuum over Na and toluene	2.3	0.2	7.3
4. Heated in *p*-dioxane	2.5	0.3	9.3

**Table 3:** Products from pyrolysis of nylon sample 4408

Sample	CO_2_	H_2_O	Cyclopentanone	Total light volatiles
				
	%	%	%	%
Untreated nylon	3.7	1.1	4.4	10.3
Treated with 1% orthophosphoric acid	7.4	1.0	0.4	12.9

## Footnotes

2 Figures in brackets indicate the literature references at the end of this paper.
